# Bread crust extract is a novel activator of aryl hydrocarbon receptor and modulator of NRF2 and NFκB in HepG2 and HCT 116 cells

**DOI:** 10.1016/j.crfs.2025.101144

**Published:** 2025-07-12

**Authors:** Anne Grosskopf, Merve Kuru-Schors, Saskia Schmidt, Jennifer Dienel, Annika Höhn, Jana Raupbach, Kristin Wächter, Conny Köhler, Tilman Grune, Gábor Szabó, Andreas Simm

**Affiliations:** a Clinic for Cardiac Surgery, University Medicine Halle, Martin Luther University Halle-Wittenberg, Halle (Saale), Germany; b Faculty of Agricultural and Environmental Sciences, University Rostock, Rostock, Germany; c Department of Molecular Toxicology, German Institute of Human Nutrition Potsdam-Rehbruecke (DIfE), Nuthetal, Germany; d German Center for Diabetes Research (DZD), Muenchen-Neuherberg, Germany; e Institute of Food Chemistry, Technische Universität Braunschweig, Braunschweig, Germany; f ZMG, University Medicine Halle, Martin Luther University Halle-Wittenberg, Halle (Saale), Germany; g DZHK (German Center for Cardiovascular Research), Berlin, Germany

**Keywords:** Aryl hydrocarbon receptor, Bread crust extract, Maillard reaction, Advanced glycation end products, NRF2, NFκB

## Abstract

The Maillard reaction describes the non-enzymatic formation of advanced glycation end products (AGEs), e.g., during thermal food processing. Studies on the mode of action and health implications of food-derived AGEs are often contradictory and lack information on active components. We use bread crust extract (BCE) as a model for an AGE-rich diet. Despite the identified AGEs and known activated signaling pathways, it is still unclear which receptors can exert the various effects described for BCE. This study investigates whether BCE can induce the aryl hydrocarbon receptor (AHR), the downstream NRF2 and NFκB signaling pathways and if this activation can be attributed to individual, free AGEs or AHR-agonists present in BCE.

HepG2 reporter cell results showed activation of AHR and NRF2 but not NFκB by BCE. However, the tested free AGEs did not show an activation. Known AHR-(pro-)agonists kynurenine (Kyn) and benzo[a]pyrene (BaP), both present in BCE, activated the reporter to a similar extent as BCE with distinct differences in target gene induction of CYP1A1, interleukin-8, heme oxygenase 1 and Manganese-superoxide dismutase. Furthermore, CYP1A1 ethoxyresorufin-O-deethylase enzymatic activity was also induced and could be modulated by AHR and NRF2-inhibition. In contrast, in HCT 116 pTRAF reporter cells, BCE activated AHR, NFκB and NRF2 and induced CYP1A1. We conclude that BCE contains potent AHR activators that influence cellular signaling activities. AHR most likely concerts cell line dependent NRF2 and NFκB-activation. The BCE effects are probably attributable to an interplay of AHR-agonists and AGEs.

## Highlights

• Bread crust extract activates the Aryl hydrocarbon receptor in reporter cell lines.
• Free advanced glycation endproducts cannot explain the activation.
• Nuclear factor erythroid-derived 2-like 2 (NRF2) activation is cell-type specific.
• Nuclear factor 'kappa-light-chain-enhancer' of B-cells is induced in HCT116 cells.
• Bread crust extract-induced cytochrome activity is modulated by NRF2 inhibition.

## Graphical abstract

**Figure:**
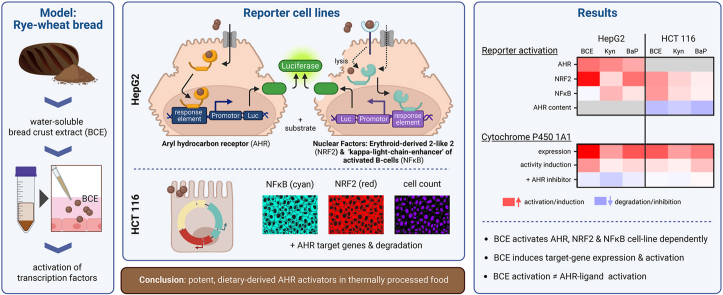

## 1 Introduction

Thermally processed foods like bakery products, coffee and roasted meat contain a wide range of bioactive components from the source materials or the production. One class of components formed due to heat treatment are Maillard reaction products (MRPs), which result from multiple-step reactions of reducing sugars with amino acids and ultimately form irreversible advanced glycation end products (AGEs). Mostly, side-chains of lysine and arginine are modified in the Maillard reaction (Snelson and Coughlan, 2019), forming a wide variety of products with different chemical properties, e.g., N-Ɛ-carboxymethyl and carboxyethyl-moieties, hydroimidazolone isomers of methylglyoxal (MG-Hs) and glyoxal (GO-Hs) as well as pentosidine, argpyrimidine and pyrraline (Rabbani and Thornalley, 2015; Sergi et al., 2021; Poulsen et al., 2013; Uceda et al., 2024). Furthermore, reactions can also lead to cross-linking of amino acids, forming more complex structures like the glyoxal-lysine dimer (GOLD) and methylglyoxal-lysine dimer (MOLD) (Poulsen et al., 2013). Endogenous AGEs and AGEs from food (dAGES) share formation mechanisms, albeit at different reaction rates and thus with different ratios of individual products (van Dongen et al., 2022). However, it is impossible to distinguish exogenously formed dAGEs from endogenously formed AGEs on the molecular level (Twarda-Clapa et al., 2022). High temperatures and oxidative conditions during food processing also foster the formation of other bioactive substances, e.g., glycoxidative amide-AGEs (Milkovska-Stamenova and Hoffmann, 2019) in milk, amino acid oxidation products like kynurenine (Liu et al., 2023) in wheat dough and acrylamides and polycyclic aromatic hydrocarbons (PAHs) among others.

Bread crust extract (BCE) is a water-soluble, AGE-rich food extract produced from rye-wheat bread and used as a model of MRP and dAGEs (Sebekova et al., 2003; Bartling et al., 2005). In general, several AGEs, including well-known representatives like Nε-(carboxymethyl)lysine (CML), Nε-(1-carboxyethyl)lysine (CEL), Nω-(carboxymethyl)arginine (CMA), Nω-(carboxyethyl)-L-arginine (CEA), Methylglyoxal-hydroimidazolone isomer (MG-H1) and Argpyrimidine (ArgPyr), were detected in BCE (Wächter et al., 2021) and other bakery products (Jost et al., 2021). Pötzsch and colleagues demonstrated that early HPLC-fractions of BCE exhibit tryptophane-derived absorption, but later fractions showed AGE-associated fluorescence and were AGE-positive in antibody-based detection, hinting towards free AGEs or small peptides. However, in a second publication, they also proved the presence of gliadins containing AGEs in BCE (Pötzsch et al., 2013a). In an MS-data set of different wheat extracts, up to 300 proteins were identified per extract with sequence stretches spanning more than 20 kDa and containing various bound AGEs (unpublished data from (Wächter et al., 2023)). Furthermore, so-called melanoidins with a size of 10–20 kDa were already described in BCE (Morales et al., 2012). Thus, BCE most likely contains free and peptide-bound dAGEs. However, quantitative data are not available for this specific model at this time.

In general, pyrraline is the most abundant AGE modification found in bakery products. It is assumed to be taken up in relatively high doses (Hellwig et al., 2024) and is actively excreted in urine following ingestion (Sergi et al., 2021). Additionally, quantitative analyses of bread varieties indicated the presence of CMA, CEA, CML, CEL, G-H1, MG-H1, pentosidine (Pent), GOLD and MOLD (Table S6) (Jost et al., 2021; Zhang et al., 2024; Scheijen et al., 2016). While global information on the effects of AGE-rich foods or extracts is available but often contradictory, results from specific AGE components are scarce (Nogueira Silva Lima et al., 2024). For example, BCE was previously shown to increase the proliferation of prostate cancer cells (Zhang et al., 2003) and to activate MAP-kinase and nuclear factor kappa B (NFκB)-signaling in murine cardiac fibroblasts (Ruhs et al., 2007), possibly by AGE-modified gliadins (Pötzsch et al., 2013a). In contrast, in human endothelial cell lines, BCE exhibited a nuclear factor erythroid 2-related factor 2 (NRF2)-activation without activation of NFκB (Wächter et al., 2021). In HeLa cells stimulated with various wheat crust extracts, the activation of NRF2-target genes Heme oxygenase 1 (HMOX1) and Glutamate-cysteine ligase regulatory subunit (GCLM) correlated with the AGE-associated fluorescence independently of acrylamide (Wächter et al., 2023). In contrast, in NFκB reporter cells, activation could be seen in a cell-type-specific but only partially AGE-dependent manner. In Jurkat-NFκB-reporter cells, total BCE showed a dose-dependent activation, and fractionated BCE showed induction in AGE-containing as well as protein fractions, while in a RAW reporter cell line, only very weak NFκB-induction was seen (Pötzsch et al., 2013b). Ex vivo, BCE protected rat aortic organ grafts from ischemia-reperfusion injury (Korkmaz-Icöz et al., 2022). One feeding study showed elevated HMGB1 levels in the lung (Bartling et al., 2007), pointing towards an pro-inflammatory response through binding of RAGE and other receptors. In contrast, induction of anti-oxidative genes in the liver and heart of mice was demonstrated in another feeding study by Wächter and colleagues (Wächter et al., 2022). It is believed that AGEs mediate all those effects via their receptor RAGE. However, fibroblasts from RAGE-knockout mice revealed at least partially RAGE-independent mechanisms of BCE, e.g., gene expression of vannin-3 and Cu/Zn-SOD, as well as phosphorylation of ERK and p38 (Leuner et al., 2012).

Furthermore, current literature is not conclusive about the exact role of RAGE as a specific receptor for AGEs (Hellwig et al., 2024). Other AGE receptors are described in the literature, including the AGE-Rs OST48, p90 and galectin-3, scavenger receptors stabilin-1 and -2, SR-AI and -BI, and LOX-1. However, signaling after binding was not observed in any of them, and they are thus considered clearance and detoxification receptors (Twarda-Clapa et al., 2022; Bahman et al., 2024; Ott et al., 2014). Curiously, Lee and colleagues reported that the aryl hydrocarbon receptor (AHR) might be involved in detecting CML (Lee et al., 2016). The AHR is a transcription factor belonging to the basic helix-loop-helix (bHLH), and periodic circadian protein, AHR nuclear translocator, single-minded protein (PAS) superfamily of transcription factors, that detects a wide variety of exogenous and endogenous small molecules and subsequently concerts context-, ligand- and cell-type-specific cellular responses (Opitz et al., 2023). The most conserved target gene of AHR-activation is the cytochrome P450 1A1 (CYP1A1), which metabolizes various endogenous substances and is also involved in detoxifying AHR ligands (Ye et al., 2019). The first AHR agonists described were PAHs and dioxins.

More recently, multiple other groups of AHR-(pro-)ligands with distinct cellular effects were described, e.g., nutritional ligands like indoles (De Juan and Segura, 2021) and various endogenous tryptophan metabolites like kynurenine, kynurenic acid (Sladekova et al., 2023) and trace-derivatives of kynurenine (Seok et al., 2018). Furthermore, there is increasing evidence, that AHR presence as well as food-derived AHR-ligands are implicated in the modulation of aging processes (Abudahab et al., 2023). In summary, this study aims to elucidate whether BCE as an AGE-rich extract can induce AHR and its target genes and how activation of AHR might relate to the known signaling pathways NRF2 and NFκB. Furthermore, we aim to identify active components of BCE by analysis of individual, free AGEs and known AHR-agonist benzo[a]pyrene and pro-agonist kynurenine.

## 2 Materials and methods

### 2.1 Bread crust extract (BCE) and R-HSA preparation

Bread crust (BC) and its extract were prepared as described (Wächter et al., 2021) with minor alterations. Shortly, a water-soluble extract was produced by mixing 250 mg BCE powder per 1 ml of PBS (Gibco, pH 7.2) and subsequent sonication. Afterward, the suspension was centrifuged at 4.800×*g* for 30 min at 10 °C and subsequently at 14,000×*g* for 30 min at 4 °C. Supernatants were filtered through a 0.1 μm PES filter, and extraction was repeated two times by adding 1 ml PBS per 333 mg BC and following the whole protocol. All extracts were mixed and then aliquoted for storage at −20 °C. AGE-modified HSA (R-HSA) was prepared as described previously for BSA (Korca et al., 2020) but with 21 days instead of 40 days of incubation. This approach resulted in an R-HSA variant with a lower degree of cross-linking, which significantly simplified handling. Nevertheless, known representatives of AGEs, e.g. CML; CEL, MG-H1 and ArgPyr could still be detected. Control HSA was prepared in the same way but without the addition of ribose to the reaction.

### 2.2 Cell lines, culture, and assays

A detailed list of chemicals used in this study can be found in the Supplemental Material.

The human hepatoma HepG2-Lucia AHR-reporter cell line was purchased from InvivoGen (hpgl-AHR, InvivoGen, San Diego, CA, USA) and cultivated in DMEM with 4.5 g/l glucose, supplemented with 10 % heat-inactivated fetal calf serum (FCS) (Capricorn Scientific GmbH; Germany), 100 μg/mL Zeocin, 100 μg/mL Normocin and 2 mM L-glutamine. HepG2 NRF2/ARE and NFκB luciferase reporter stable cell lines were purchased from Signosis (SL-0046/SL-0017, Signosis, Santa Clara, CA, USA) and cultivated as described before for AHR-Reporter, except for 100 μg/ml Hygromycin B Gold as selection antibiotic.

HCT 116 pTRAF reporter cells, carrying three distinct fluorescent reporters for NRF2 (mCherry), NFκB (TFP) and HIF1α (YPet), were cultivated in McCoy's 5A with 10 % FCS, 1 % PenStrep, 1 % and 200 μg/mL Hygromycin B. In all cell lines, selection antibiotics were not used in experiments but only during propagation. For the enzyme activity assays, non-reporter HepG2 (HEP-G2, ACC 180) and HCT 116 (HCT-116, ACC 581) cells from the DSMZ (Braunschweig, Germany) were used. The identity of all cell lines except for HCT 116 pTRAF was verified by STR-analysis (Eurofins Genomics). All cell lines were incubated at 37 °C in a humidified atmosphere at 5–10 % CO_2_.

#### 2.2.1 HepG2 reporter cell assays

For assays, all HepG2 reporter cells were seeded at a density of 66,000 cells/cm^2^ to ensure sub-confluence at the start of the experiment. Then, they were grown for three days and harvested by trypsinization with Trypsin/EDTA in PBS for 5 min at room temperature (RT). Subsequently, 20,000 cells per well were plated in a white-wall, white-bottom 96-well plate in 180 μl of phenol-red free DMEM (assay medium). The next day, 20 μl of diluted compounds of interest or controls in the assay medium were added. The plate was shaken for 10 s at 500 rpm and subsequently incubated for the indicated time of induction and/or inhibition. Luciferase solutions were frozen and transferred to RT 45 min before the experiments. For the AHR reporter cells, 20 μl of cell supernatant were transferred into a new white 96-well plate with 50 μl of QUANTI-Luc Gold luciferase substrate, and signals were measured in a CLARIOstar Plus device (BMG Labtech, Ortenberg, Germany, Firmware 1.32, Software 5.6.1). Settings are found in Table S1. The medium was aspirated for the NRF2/ARE, and NFκB luciferase reporter stable cell lines and 100 μl of fresh assay medium were supplied. Then, 100 μl of Bright Glo luciferase substrate was added, shaken for 10 s and then the plate was incubated for 2 min at RT to facilitate cell lysis before measurement in the CLARIOstar plus device (Table S1). As positive controls, the following substances were applied: 1.8 μM FICZ for the HegG2 AHR-reporter, 5 μM tBHQ for the NRF2-reporter, and 20 ng/ml TNFα for the NFκB-reporter as proposed by the manufacturer. TNFα was additionally evaluated for the absence of cytotoxicity and maximal activation (see Fig. S1). Compounds of interest used were BCE, Kyn, and BaP. All results were normalized against their respective medium controls and presented as fold change (FC) signals. Cell viability and metabolic activity determination were routinely performed during experiments utilizing the cell titer blue assay (see Supplemental Methods).

#### 2.2.2 HCT 116 reporter cell assays

The activation of NRF2 and NFκB in HCT 116 pTRAF cells was assayed as described previously (Raupbach et al., 2023). HCT 116 pTRAF cells were stimulated for 24h with positive controls 10 μM FICZ for AHR, 10 ng/ml TNFα for NFκB, and 2 μM Auranofin for NRF2 activation. As compounds of interest, 2.5 %, 5 % and 10 % BCE, including a PBS control, 150 μM Kyn, and 250 and 500 nM BaP, including a DMSO control, were used.

### 2.3 qPCR

#### 2.3.1 Primer design

QRT-PCR primers were designed with Primer-BLAST unless indicated otherwise and tested in silico for specificity. Production was done by Eurofins (Ebersberg, Germany) or Metabion (Planegg, Germany). Primers were dissolved in nuclease-free H_2_O with a final 100 pmol/μl concentration and stored at −20 °C. Additional primer information can be found in Tables S2 and S3 and Fig. S2.

#### 2.3.2 RNA isolation

Cells were seeded in flat bottom 6-well plates (TPP; Switzerland, 92006) at 44.300 cells/cm^2^ and stimulated as indicated the next day. Subsequently, cells were lysed in 1 ml TRIzol reagent after medium aspiration and samples were transferred to 1.5 ml reaction tubes. Then, 200 μl of chloroform puriss.p.a. (Sigma-Aldrich, 32211-1L-M) were added, and samples were shaken for 30 s, followed by an incubation at RT for 3 min. Subsequently, samples were centrifuged at 12,000×*g* for 15 min at 4 °C. The formed aqueous phase was then transferred to a 1.5 ml reaction tube containing 500 μl 2-propanol (Sigma-Aldrich, 33539-M) and mixed by inverting. Following RNA precipitation at −20 °C for 30 min, another centrifugation step was done at 12,000×*g* for 15 min at 4 °C. The resulting RNA pellet was washed twice with 75 % ethanol, mixed from absolute ethanol (Merck, Supelco, 1.00983.1011) and HPLC-grade water (Chemsolute, 418.1000), through spinning at 8000×*g* for 5 min at 4 °C. Finally, the RNA pellet was dried and dissolved in nuclease-free H_2_O. RNA content and purity were determined by Nanodrop ND-1000 (Thermo Fisher Scientific) according to the absorbance (A) at 280 nm and the ratios A_260_/A_280_ and A_260/_A_230._ Samples were only accepted for qPCR if A_260_/A_280_ ≥ 1.9 and A_260/_A_230_ ≥ 1.4. For lower A_260/_A_230_ ratios, a sample cleanup was done with the RNeasy MinElute Cleanup Kit (Quiagen, ID: 74204) following the manufacturer's protocol.

#### 2.3.3 cDNA synthesis

One μg of RNA was used for cDNA synthesis with 1 μg random hexamers from a stock solution of 500 μg/ml in H_2_O (Metabion, Planegg, Germany) per μg of RNA as follows. The samples were diluted in 15 μl of H_2_O in 0.5 ml reaction tubes, and reverse transcription (RT) was carried out in an Eppendorf Mastercycler Gradient. First, the samples were heated for 5 min at 70 °C, cooled at 4 °C for 5 min and spun down shortly. Subsequently, 12.5 nmol dNTP mix (New England Biolabs, N0447S), 200 units M-MLV Reverse Transcriptase (RNase H Minus, Point Mutant, Promega, M3682) and accompanying 5x reaction buffer (50 mM Tris-HCl (pH 8.3), 75 mM KCl, 3 mM MgCl_2_, 10 mM DTT) were added. The reaction volume was set to 25 μl. After gentle mixing, the reverse transcription was finished with the following settings: 10 min at 21 °C, 50 min at 42 °C, and 15 min at 70 °C in the same cycler. Finally, the cDNA was diluted 1:8 in nuclease-free H_2_O for qPCR.

#### 2.3.4 qRT-PCR

The qPCR was carried out in a total volume of 10 μl, comprised of 1 μl diluted cDNA, 1 pmol of each forward and reverse primer, 5 μl of 2x SsoAdvanced Universal SYBR Green Supermix (Bio-Rad, Hercules, CA, USA, 1725271) and H_2_O. Samples were prepared using technical triplicates, and no-template controls (NTCs) were included for each primer pair. The qPCRs were prepared in white hard-shell, thin wall, low profile skirted 96-well plates (Bio-Rad, #HSP9655) sealed with clear PCR-foil (Sarstedt, Nümbrecht, Germany, 5.1999). The qPCR was done in a CFX-connect Real-Time PCR Detection System (Bio-Rad) operated with CFX manager software version 3.1.1517.0823 (Table S4). Products were always analyzed for their specific melt peaks, and samples were excluded if any deviations ≥0.5 °C occurred.

The Cq-values were determined by the CFX manager software and subsequently exported to Microsoft Excel for ΔΔCq-normalization. Single technical replicates or whole samples were excluded from analysis whenever Cq values differed more than 0.5 cycles. Large ribosomal subunit protein uL10 (RPLP0) was utilized as a housekeeping gene for all runs. Normalization was carried out against the housekeeping gene and then against the treatment controls; in the case of BCE PBS, for all other substances, the respective DMSO control using the 2^−ΔΔCq^-method.

### 2.4 Protein harvest, SDS-page, western blot for AHR-degradation

HepG2 and HCT 116 pTRAF cells were seeded as described for RNA isolation. To monitor AHR-degradation, cells were sequentially stimulated for 2 h, 4 h, 6 h, 8 h and 24 h as described by Grosskopf and colleagues (Grosskopf et al., 2021) with indicated compounds and 24 h with PBS and DMSO as controls. In short, protein harvest was done in 50 μl Triton protein lysis buffer (50 mM Tris-HCl, 150 mM sodium chloride, 0,5 % (w/v) sodium deoxycholate, 0,5 % (v/v) SDS, 1 % (v/v) Triton X-100, 1x Protease inhibitor cocktail, 5 mM sodium orthovanadate, ∼25 U Benzonase Nuclease (Merck Millipore, 70664-3) per 5 ml buffer, pH 7.4) using a sterile cell scraper (TPP99002). At the same time, the plate was placed on wet ice. After transferring in 1.5 ml reaction tubes and incubating on ice for 30 min, the samples were centrifuged for 10 min at 8000×*g* and 4 °C. Finally, the supernatant was transferred to a fresh 1.5 ml reaction tube. Protein concentration was determined using the BCA Protein Assay Kit (Thermo Scientific) Microplate procedure described by the manufacturer. For the SDS- PAGE, 25 μg of protein were loaded with sample buffer (200 mM Tris/HCl pH 6.8, 5 % (v/v) β-mercapto ethanol, 10 % (v/v) glycerol, 2 % (v/v) SDS, bromophenol blue) on a self-cast, 1.5 mm thick, 10 % acrylamide gel with 4 % stacking gel. The PAGE was run at 80 V for 10 min and at 120 V until the 10 kDa Marker band (PageRuler Plus prestained, 26619) reached the bottom of the gel. The samples were then transferred onto a 0.2 μm nitrocellulose membrane by either turbo blot (HCT 116) or tank blot (HepG2). The Bio-Rad Trans-Blot Turbo Transfer System was used with the standard SD protocol (25V, 1 A, 30 min) for turbo blot. For tank blot, the parameters were 120 V and 4 °C for 90 min. As loading control, membranes were stained with total protein staining (Revert™ 700 Total Protein Stain, 926–11011, LI-COR, Lincoln, NE, USA), imaged on an Odyssey CLx Imaging System (LI-COR) at 700 nm and destained according to manufacturer's protocols. Blocking was done in 5 % (w/v) BSA in TBS-T (ROTI®Fair TBS 7.6 tablet in 500 ml MQ water with 0.1 % (v/v) Tween 20) for 1 h at RT followed by the incubation with primary antibody overnight at 4 °C. The next day, the blot was washed 5x for 5 min in TBS-T and incubated with a secondary antibody for 1 h in TBS-T at RT in the dark. After the final washing, signals were detected using the Odyssey CLx system and quantified using Image Studio Ver. 5.2. All signals were normalized using the respective total protein stains, and ratios were built by setting the 0 h time-points to one to report a relative decrease in protein concentrations.

### 2.5 Ethoxyresorufin-O-deethylase (EROD) activity assay

CYP1A1 enzymatic EROD activity was measured as described before (Behrens et al., 1998) with adaptations to the cell types used. HepG2 and HCT 116 cells were seeded into a 96-well plate at 94,000 cells/cm^2^ and incubated overnight at 37 °C, 10 % CO2. The following day, cells were stimulated with compounds of interest or controls for 24 h. Then, the supernatant was discarded, and the cells were washed with PBS. Subsequently, 9 μM Dicoumarol and 8 μM 7-Ethoxyresorufin were added for 20 min in phenol red-free DMEM without the addition of FCS. Finally, fluorescence was measured at Ex: 544 nm, Em: 590 nm with 5 nm bandwidth at a TECAN M1000 device (Männedorf, Switzerland).

### 2.6 Statistics

For all experimental results presented, counts of biologically independent experiments (N = ) and technical replicates within any given experiment (n = ) are reported. Statistical evaluation was done in GraphPad Prism version 9.5.1.733 for Windows (GraphPad Software, San Diego, California USA (http://www.graphpad.com)).

#### 2.6.1 Normality-testing

To delineate whether results were sampled from a Gaussian distribution, the Shapiro-Wilk test was carried out before further statistical analysis. The alpha was set to 0.01. In the case of normal distribution, parametric tests were utilized.

#### 2.6.2 Significance testing and effect sizes

For all reporter cell, qPCR, and EROD results, unpaired multiple Welch-t-tests not requiring homogeneity of variance were calculated. The results were not corrected for multiple testing because effects were only compared to their respective controls or, in the case of inhibitor use, to their effectors. For AHR-degradation, one-sample t-tests with a hypothetical mean of one were calculated. Reported effect sizes represent Cohen's d calculated using mean, standard deviation (SD), and sample sizes (biological replicates).

#### 2.6.3 EC50 and decay-analysis

The EC_50_ was calculated via the agonist vs. response (three parameters) analysis. A one-phase decay with robust regression and medium stringency was used for decay-analysis. Result parameters, i.e., best-fit values, the goodness of fit and constraints for the EC_50_ and decay analysis, are presented in the supplement.

## 3 Results and discussion

### 3.1 BCE induces AHR- and NRF2- but not NFκB-dependent luciferase expression in HepG2 reporter cells

**Fig. 1:**
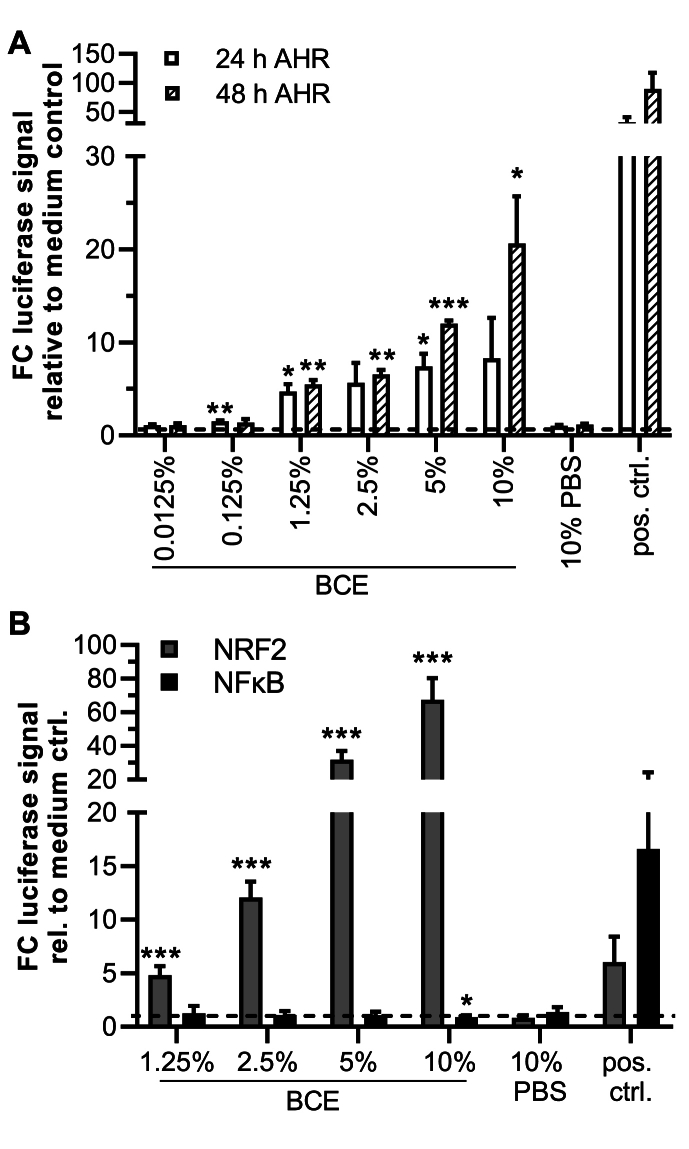
BCE-mediated luciferase signal induction in HepG2 reporter cells. Data are presented as mean fold-change above media controls ±SD. p-values ∗ <0.05, ∗∗ <0.01, ∗∗∗ <0.001. A: AHR-dependent Luciferase signals are induced in a dose-dependent manner by BCE after 24 h and to a greater extent after 48 h of incubation. N = 3, n = 3, Pos. ctrl.: 1.8 μM FICZ. B: NRF2- but not NFκB-HepG2-reporter cells showed dose-dependent induction of luciferase signals by BCE. N = 7, n = 3. Pos. ctrl.: 5 μM tBHQ, 20 ng/ml TNFα. FC: fold change, Pos. ctrl.: positive control(s).

To clarify whether BCE can induce AHR, a commercially available HepG2 luciferase reporter cell line for AHR was analyzed for its activation in the presence of increasing percent (v/v) of BCE in cell culture medium for 24 h and 48 h. Indeed, 0.125 %–5 % BCE induced a significant AHR-mediated luciferase signal of 1.5- to 8-fold above medium control after 24 h (Fig. 1A). After 48 h, the induction was lost at 0.125 %, comparable in magnitude for 1.25 % and 2.5 % and more pronounced at higher concentrations with a mean induction of 12-fold at 5 % BCE and 20.6-fold at 10 % BCE. The EC_50_ values for luciferase induction determined were 1.12 % BCE (CI: 0.58-1-99) at 24 h and 3.36 % BCE (CI: 2.45–4.64) at 48 h of induction (Table S5). The AHR reporter results indicate that BCE can evoke a sustained AHR-mediated luciferase expression and might thus be able to promote activation of AHR and target gene expression from 0.125 % to 10 %. Similar results were recently published for HepG2 and other cell lines when incubated with varieties of coffee but not tea or cocoa (Ishikawa et al., 2014; Toydemir et al., 2021; Chapkin et al., 2021). Hence, it might be a common property of plant-derived, heated, AGE-rich extracts to activate AHR, at least in intestinal and liver cells. In contrast to BCE, human serum albumin modified by ribose incubation (R-HSA), used as another model of AGEs, and the control HSA did not induce the AHR reporter cells (data not shown), which indicates that a single type of AGE-modified protein is not able to activate AHR.

To elucidate if AHR-activation by BCE occurs in concert with the previously described activation of Nuclear factor erythroid 2-related factor 2 (NRF2) or Nuclear factor kappa B (NFκB) transcription factors, respective HepG2 reporter cells were also investigated. For NRF2, we could show a significant, dose-dependent induction of luciferase after 24 h of incubation in the same concentration range as for AHR (Fig. 1B). While the FC induction was similar for both pathways at lower concentrations, the NRF2-reporter showed a mean 32- and 67.4-fold induction at 5 % and 10 % BCE, respectively. However, since the reporters use different luciferase systems, whether this is a reporter or a biological effect is not deductible. In contrast to AHR and NRF2, no activation of the NFκB reporter was observed in the applied concentration range of BCE. In contrast, 10 % even showed a suppression effect for NFκB (effect size: 2.33). The positive control, 20 ng/ml tumor necrosis factor alpha (TNFα), showed a mean signal-induction of 16.6-fold. Thus, BCE activates AHR and NRF2 dose-dependently but does not activate NFκB. These results align with previous findings in other cell types, e.g., endothelial cell lines, where BCE induced NRF2 and showed gene regulation patterns prompting to anti-oxidative properties (Wächter et al., 2021). Again, coffee was also reported to activate AHR- and NRF2 in vitro (Toydemir et al., 2021). However, from our results with the HepG2 luciferase reporter cell line, it is unclear whether the activation of AHR and NRF2 is independent. In the literature, both NRF2-mediated AHR activation and AHR-dependent NRF2-induction are described (Bahman et al., 2024). Identifying specific BCE compounds responsible for the reported activation would be valuable to delineate the transcription factor responses.

### 3.2 Modulation of AHR, NRF2 and NFκB activity facilitated by BCE cannot be modeled by free AGEs or known AHR-agonists Kyn and BaP

To further elucidate the bio-active compounds and clarify whether AGEs are involved in AHR-activation, all HepG2 reporter cell lines were incubated with a selection of eight amino acid modifications (free AGEs), namely N-ω-carboxymethyl-L-arginine (CMA), N-ω-carboxyethyl-L-arginine (CEA), N-ε-carboxymethyl-L-Lysine (CML), N-ε-carboxyethyl-L-Lysine (CEL), Argpyrimidine (ArgPyr), Glyoxal-hydroimidazolone isomer (G-H1), Methylglyoxal-hydroimidazolone isomer (MG-H1), and pyrraline (Pyr) and three cross-linked amino acids, pentosidine (Pent), Glyoxyl-derived lysine dimer (GOLD), and Methylglyoxyl-derived lysine dimer (MOLD) already described to be present in bakery products (Wächter et al., 2021; Jost et al., 2021). Each AGE's concentration range was 0.025 μM–250 μM for a 24 h incubation period. The incubation with AGEs did not result in a significant luciferase induction for any of the reporters and concentrations tested, nor did the incubation with cross-links (Fig. S3 A-F). The theoretical content of AGEs in BCE derived from quantitative data of bread varieties resulted in possible nM to lower μM concentrations (see Table S6). Even if the extraction efficiency would be as low as ten percent, the concentrations of AGEs used in the reporter assays are plausible in the context of exposure to dAGEs and nicely match the expected contents of AGEs in bread.

In summary, none of the investigated AGEs mirrored the luciferase induction of AHR and NRF2 in the HepG2 reporter cells with BCE. On the one hand, it might be that free AGEs are not substrates of AHR nor bind receptors activating NRF2 or NFκB. On the other hand, these results could also stem from low influx rates of free AGEs into cells due to limited bioavailability (Jansen et al., 2023). In the case of CML and pyrraline, several studies indicated absorption of the free and protein-bound products after ingestion (Hellwig et al., 2009, 2024). Since results from free AGEs did not explain the reporter responses detected, BCE was analyzed for known AHR-(pro-)agonists, of which the presence of kynurenine (Kyn) and benzo[a]pyrene (BaP) could be confirmed by slot-blot analyses of 0.5 % and 1 % BCE. Both agonists were absent in R-HSA and the respective native HSA control (Fig. S4).

**Fig. 2:**
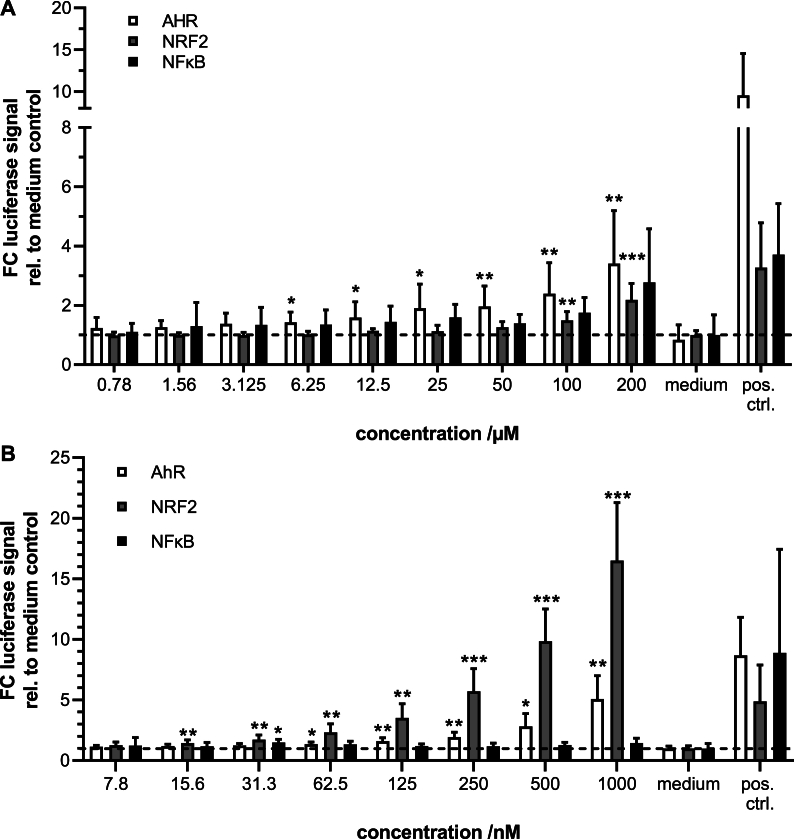
Induction of luciferase expression in HepG2 reporter cells by known AHR-(pro-)agonists. N = 5–8, n = 3. p-values ∗ <0.05, ∗∗ <0.01, ∗∗∗ <0.001. A: Kyn is able to significantly and dose-dependently induce AHR-dependent and to a lesser extend NRF2-dependent luciferase expression at 24 h of incubation. B: BaP significantly induces AHR from 62.5 nM, NRF2 from 15.6 nM and at 31.3 nM also NFκB-dependent luciferase expression in HepG2-reporter cells. FC: fold change.

To investigate whether AHR agonists could be responsible for reporter activation by BCE, both were tested in all three HepG2 reporter cell lines. Kyn was used in a concentration range between 0.01 μM and 200 μM and showed a significant AHR-mediated luciferase induction above 6.25 μM when incubated for 24 h (Fig. 2A). For the NRF2-reporter, a significant induction was only seen in the high concentrations 100 μM and 200 μM and was small with 1.5- fold (effect size: 0.21) and 2.1 fold (effect size: 0.29). At those concentrations, there was a trend towards induction in the HepG2 NFκB-reporter cells with 1.8 fold (effect size: 0.12) and 2.7 fold (effect size: 0.36). However, the results were not statistically significant, possibly due to the higher biological noise generally present in the NFκB-reporter cells and the small effect sizes. Although Kyn can induce both HepG2 AHR- and NRF2- and possibly also NFκB reporter cells, the necessary concentrations and the measured fold changes are not comparable with the responses seen in BCE. A Kyn-Elisa was used to determine concentrations in BCE, which resulted in values below the limit of quantification (data not shown). Yilmaz and Gökmen determined a Kyn-concentration of 0.144 mg per kg dry weight in bread (Yilmaz and Gokmen, 2018). This would approximate 70 nM Kyn in BCE, provided the extraction is complete and there is no crust-to-crumb gradient. Thus, it is unlikely that Kyn is the main active compound in BCE. However, the results do not exclude a synergistic influence of Kyn on the total TF activation. Additionally, BCE could contain trace-extended aromatic condensation products (TEACOPs), which are derivatives of Kyn and activate AHR at picomolar concentrations (Seok et al., 2018). TEACOPs could then also facilitate the activation of other TFs through AHR.

In contrast, BaP incubation, in a tested concentration range of 7.8 nM to 1 μM, lead to a significant reporter cell activation at more than 100-fold lower concentrations than Kyn. For the AHR-reporter, 1.35-fold induction was found already at 62.5 nM with an increase to 5-fold in 1 μM. In the NRF2-reporter, higher fold changes and a more substantial increase were observed with a significant activation already at 15.6 nM, a 2.3-fold induction at 62.5 nM and 16.5-fold at 1 μM (Fig. 2B). For the NFκB-reporter statistically significant induction by BaP was found at only 31.3 nM (FC 1.5, effect size: 2.38). Concentrations above 1 μM BaP were not analyzed due to cytotoxicity as determined by reduced metabolic activity in the CTB assay (Fig. S5). In general, the reporter activation by BaP up to 1 μM mirrored the induction seen in BCE.

**Fig. 3:**
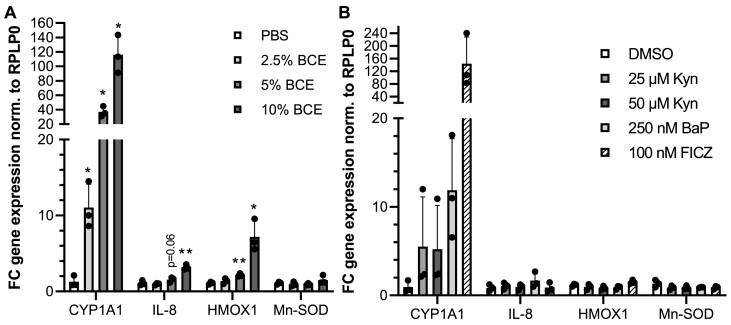
Target gene induction in HepG2 cells upon stimulation with BCE, Kyn and BaP. N = 3, n = 3. P-values ∗ <0.05, ∗∗ <0.01. A: BCE strongly induces the AHR-target gene CYP1A1, Heme oxygenase 1 (HMOX1) and interleukin 8 (IL-8) but not Manganese superoxide dismutase (Mn-SOD). B: Target gene activation of AHR-(pro-)agonists BaP, Kyn and FICZ.

We investigated the target-gene expression of wild-type HepG2 cells to validate the reporter induction using qRT-PCR. As suspected, 2.5 %, 5 % and 10 % BCE significantly induced the AHR target gene CYP1A1 (Fig. 3A). The maximal mean induction detected was 116-fold in 10 % BCE, and the increase of induction between 2.5 % and 5 % was 3.3, between 5 % and 10 %, 3.2-fold. In line with previous results (Wächter et al., 2021), 5 % and 10 % BCE also induced Heme oxygenase 1 (HMOX1), an NRF2 target gene. Furthermore, IL-8, which is a target gene that significantly contributes to both AHR and NFκB signaling is also significantly induced in 10 % and potentially also in 5 % BCE (p-value: 0.06. effect size: 0.99) albeit with no significance due to the number in replicates. The extent of induction is much lower than for CYP1A1. Surprisingly, HMOX1 was not induced by BaP (Fig. 3B), although the NRF2-reporter showed significant luciferase induction. The seemingly contradictory lack of induction of HMOX1 in BaP-treated NRF2 reporter could be explained by the repressive action of BACH1, which blocks the HMOX1 promotor for NRF2 (Reichard et al., 2007). Alternatively, it would also be possible that HMOX1 is not directly regulated by NRF2 but via BCE-mediated ERK-activation and, subsequently, AP-1 (Medina et al., 2020). However, this result indicates that the effect of BCE cannot solely be attributed to BaP and warrants further investigation. In summary, while the target gene expression results could affirm AHR and NRF2-activation by BCE, none of the analyzed individual compounds, such as AGEs, Kyn or BaP, sufficiently explained the presented results.

### 3.3 NRF2 and NFκB activation by BCE is cell-type-specific, while AHR is ubiquitously activated

**Fig. 4:**
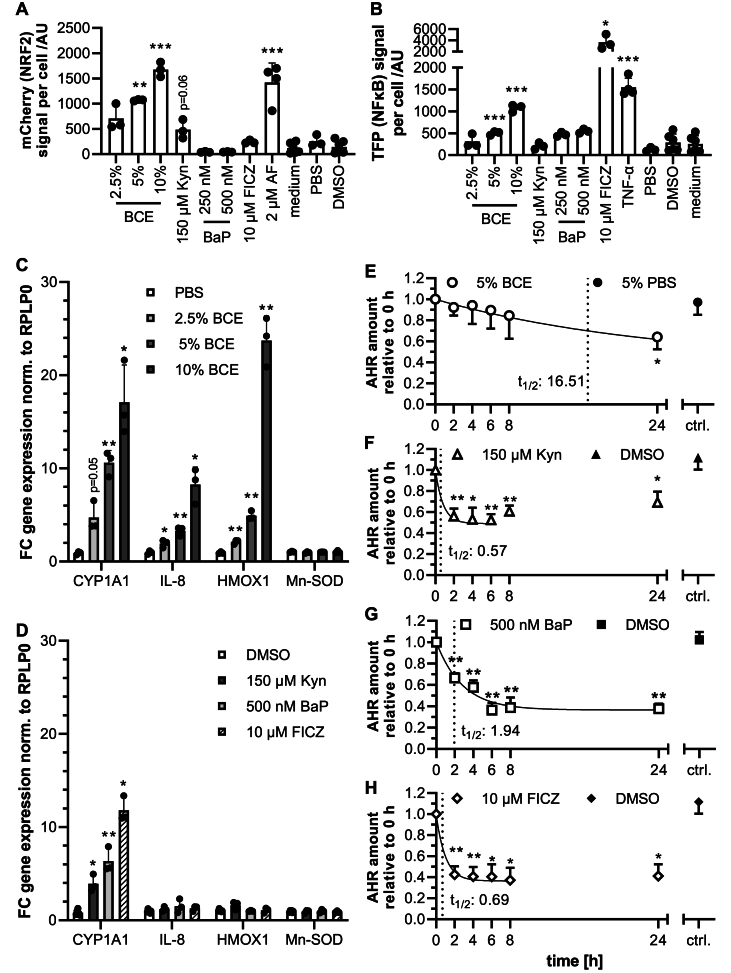
AHR activation and induction of NRF2 and NFκB reporter-fluorescence by BCE, Kyn and BaP in HCT 116 reporter cells. A: Significant NRF2-dependent mCherry induction was reported for all concentrations of BCE, for Kyn (N = 3) and for the positive control, 2 μM auranofin (N = 4) (AF) after 24h of stimulation. N = 3–6 for controls. P-values against solvent controls (PBS/DMSO) ∗ < 0.05, ∗∗∗ < 0.001. B: Induction of NFκB reporter fluorescence (TFP) was seen in 5 %–10 % BCE and the controls FICZ and 10 ng/ml TNFα (all N = 3) after 24 h incubation. N = 3–6 for DMSO and medium-controls. P-values: ∗∗∗ <0.001. C&D: All tested inducers showed significantly increased CYP1A1 expression as a result of AHR-activation. Only BCE induced HMOX1 and interleukin IL-8 in a dose-dependently. Mn-SOD was not induced. N = 3, n = 3. E–H: AHR-degradation half-life and dynamics after activation were analyzed by anti-AHR Western blot for (E) 5 % BCE, (F) 150 μM Kyn, (G) 500 nM BaP, and (H) the positive control FICZ at 2 h, 4 h, 6 h, 8 h and 24 h of incubation. All substances showed varying extents of AHR-degradation, with the longest half-life (t_1/2_) in BCE. N = 3. P-values from one-sample t-tests, ∗ <0.05, ∗∗ <0.01.

Since cell-type specific activation of signaling pathways by BCE was previously reported for NRF2 and NFκB in Ea.hy926 and HeLa cells (Wächter et al., 2021), Jurkat and RAW reporter cells (Pötzsch et al., 2013b) and macrophages, we investigated a second reporter cell line for all three pathways. The HCT 116 pTRAF reporter cell line can show NRF2 and NFκB activation by fluorescence expression of TFP and mCherry. In contrast to the hepatoblastoma HepG2 cell line, HCT 116 was derived from a colon carcinoma. When stimulated with 5 % and 10 % BCE, HCT 116 reporter cells showed a significant induction of both NRF2 and NFκB-dependent fluorescence (Fig. S6), while at lower concentrations, only the NRF2-reporter showed a significant induction (Fig. 4A and B). The dose-dependent and apparent activation of NFκB contrasts the results from the HepG2 reporter cell lines and hints towards a cell-type specific activation of signaling pathways and transcription factors, especially of NFκB. The effect of the free and protein-bound AGEs CML and pyrraline on HCT 116 pTRAF cells was studied recently (Raupbach et al., 2023). While free compounds did neither activate NRF2 nor NFκB, casein-bound pyrraline was able to activate NRF2. From previous results, polypeptide- or protein-bound AGEs, e.g., gliadins in BCE, likely contribute to the observed effects. The results presented in this study were acquired using free AGEs on the reporter cells, which showed no activation, thus supporting this hypothesis.

Regarding AHR agonists in BCE, the weak induction of NRF2 at high concentrations of Kyn was also detected in HCT 116 cells, while BaP did not induce the fluorescence reporters at all. Since the HCT 116 reporter does not have a reporter function for AHR activation, we assessed this indirectly via target-gene expression (Fig. 4C and D) and analysis of AHR-degradation (E-H) following stimulation. Indeed, a significant induction of the AHR target gene CYP1A1 could be seen for all tested compounds after 24 h of incubation. The increase from 2.5 % to 5 % BCE led to a 2.2-fold increase in the mean induction. The increase from 5 % to 10 % only led to a 1.6-fold increase in induction, pointing towards a plateau in activation. Furthermore, only BCE induced the NRF2 target gene HMOX1 and IL-8 significantly. In line with the HCT 116 NRF2 and NFκB reporter results, BaP did not induce other tested target genes than CYP1A1 in the HCT 116 cells (Fig. 4D). Generally, the magnitude of induction of CYP1A1 expression for the chosen concentrations is comparable to the induction with BCE. These results corroborate the ones presented for the AHR and NRF2 HepG2 reporter cells. An interesting data point to discuss is the target gene induction of IL-8 in HepG2 and HCT 116 but at different magnitudes (∼4-fold vs. ∼8-fold). This IL-8 expression is a response to a non-canonical AHR activation involving RelB or NFκB (Denison and Faber, 2017). Dependent on the protein repertoire in the cells, this mechanism might lead to the activation of NFκB (Chan et al., 2017) detected in HCT 116 cells but not in HepG2.

The analysis of AHR degradation dynamics after stimulation with 5 % BCE revealed a considerably slower dynamic (t1/2: 16.51 h) than any tested single AHR agonist (t_1/2_ between 0.6 and 1.9 h). However, the plateaus resulting from the degradation are comparable with 0.364 for the positive control FICZ, 0.364 for BaP and 0.399 for BCE. Only Kyn-induced degradation (Fig. 4F) was more transient, with a plateau of 0.49 and a beginning increase of AHR content after 6 h. The different kinetics of BCE and the other compounds indicate that AHR's distinct mode of action in response to BCE differs from that of known individual agonists. Decay analysis, on the other hand, revealed that the magnitude of genomic AHR-signaling indicated by the plateau of residual AHR after activation is similar in BCE and the other tested compounds (Table S7), rendering it a genuine AHR-activator.

### 3.4 BCE induces CYP1A1 enzyme activity via the AHR, and induction can be modulated by NRF2-inhibition

**Fig. 5:**
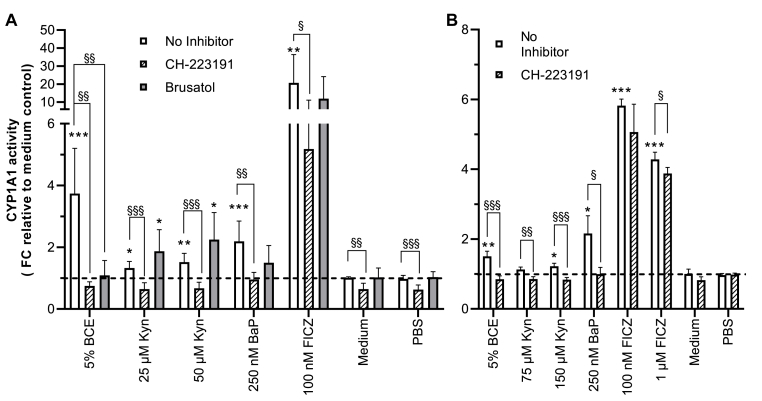
Induction of CYP1A1 activity by BCE, Kyn and BaP in HepG2 and HCT 116 cells determined by the EROD assay after 24 h of induction. A: HepG2 cells, N = 6, n = 3. B: HCT 116 cells, N = 4, n = 3. The AHR antagonist CH-223191(1 μM) and NRF2-inhibitor Brusatol (30 nM) were used in HepG2-cells to test for AHR-specificity and NRF2 interplay. Denotion of p-values from unpaired t-tests against medium controls ∗ <0.05, ∗∗ <0.01, ∗∗∗ <0.001, p-values from unpaired t-tests of inhibition against effects § < 0.05, §§ < 0.01, §§§ < 0.001.

To confirm that activation of AHR leads to modulation of CYP1A1 drug-metabolizing activity, we conducted an EROD assay after stimulation with BCE and agonists for 24 h. In HepG2 cells, CYP1A1 activity was significantly induced for all tested compounds (Fig. 5A). Inhibition of AHR-activation by addition of 1 μM of the inhibitor CH-223191 1 h before stimulation resulted in a CYP1A1 activity below basal levels except for the positive control FICZ, where the induction could not be inhibited by CH-223191, probably due to ligand-selective antagonism (Ondrova et al., 2023). Under control conditions, the inhibitor also diminished the basal activity of CYP1A1. Interestingly, when 30 nM of Brusatol, an NRF2 inhibitor, was added, a differential pattern for inhibition could be seen. For 5 % BCE, CYP1A1 induction was inhibited to a similar level as CH-223191. For Kyn, in contrast, there was no inhibition of the induction, but a trend to a further increase in activity was seen. While CYP1A1 expression stayed stable with increasing Kyn-concentration from 25 μM to 50 μM, as was seen before in the qRT-PCR results (3.2), its activity increased upon NRF2-inhibition. For BaP, inhibition of the CYP1A1 activity was non-significant upon NRF2-inhibition. However, an apparent reduction is visible. Of interest was that Brusatol could not further reduce basal CYP1A1 activity. Detection of the CYP1A1 enzyme activity in HCT 116 cells showed similar results to HepG2 (Fig. 5B). The magnitudes of induction were generally smaller except for BaP, where both cell lines showed a similar magnitude of induction.

Differential activation of TFs could be mediated by intracellular differences in protein repertoire and the expression of receptors. Regarding receptors attributed to AGE-binding, HepG2 and HCT 116 cells express most of the receptors at the RNA level except for Stabilin-1 in HCT 116 (Human Protein Atlas: proteinatlas.org (Jin et al., 2023)). On the protein level, RAGE (Raupbach et al., 2023) and AGE-Rs one to three were detected consistently in both lines. However, differences in the presence of scavenger receptors (SR) and stabilins were found (Shi et al., 2018; Geiger et al., 2012; Frejno et al., 2017; Shiromizu et al., 2013). It cannot be ruled out that this discrepancy in receptor expression contributes to the TF activation. The most promising difference would be in SR-AI, which seems only present in HCT 116 cells and was previously described as recognizing OVA modified by pyrraline (Heilmann et al., 2014). Still, the in vivo relevance of these receptors for AGE-binding is unclear, and molecular mechanisms would need further elucidation. For the detection of xenobiotics, two other potential receptors need to be considered: the pregnane X receptor (PXR) and the constitutive androstane receptor (CAR) (Mackowiak and Wang, 2016). While CAR is expressed in both investigated cell lines, they differ in the expression of PXR (Jin et al., 2023). HepG2 cells lack the pregnane receptor, and it could be shown previously that the cellular response to BaP is altered depending on the presence of PXR (Reed et al., 2020).

Some limitations of the results need to be acknowledged. First, the reporter systems employed are optimized for robust activation but may lack sensitivity for detecting weak or suppressive effects. Furthermore, for NRF2 and NFκB, evidence of activation is currently restricted to the reporter signals and RNA expression data. While these results suggest transcriptional activation, they do not necessarily imply functional consequences at the protein level. Thus, further experiments on the pathway activation in native cell lines would be of benefit in future studies to substantiate the reporter-cell findings.

## 4 Conclusions

In summary, BCE can activate AHR in HepG2 and HCT 116 cell lines and induce NRF2 and NFκB in a cell line-dependent manner and magnitude. This biological activity could not be solely explained by free AGEs or the known AHR-agonists BaP or Kyn present in BCE. The AHR-(pro-)agonists had distinct dynamics and target gene expression profiles that discriminated them from responses to BCE stimulation. This indicates, in conclusion, that free AGEs are not the AHR-activators in BCE and that further research is needed to determine the active compounds, e.g., protein-bound AGEs or small molecules, and their respective receptors. BCE also induced CYP1A1 activity in both cell lines, which could be inhibited by both AHR- and NRF2 antagonists in HepG2 cells, indicating interdependency of NRF2- and AHR-activation. While the non-toxic action of dietary AHR ligands from vegetables is well studied and often discussed in the field, stable dietary AHR ligands from thermally processed foods are underrepresented in current research. However, our study showed that extracts from such foods contain potent AHR activators that modulate cellular signaling in a complex cross-talk between AHR, NRF2 and NFκB.

## CRediT author statement

Anne Grosskopf: Conceptualization, Methodology, Formal Analysis, Writing – Original Draft, Visualization, Supervision, Project administration; Merve Kuru-Schors: Investigation, Writing - Review & Editing, Formal Analysis, Validation, Visualization; Saskia Schmidt: Investigation, Writing - Review & Editing; Jennifer Dienel: Investigation, Writing - Review & Editing; Annika Höhn: Writing - Review & Editing, Supervision; Jana Raupbach: Writing - Review & Editing, Supervision; Kristin Wächter: Resources, Methodology, Writing - Review & Editing; Conny Köhler: Resources, Writing - Review & Editing; Tilman Grune: Resources, Writing - Review & Editing, Supervision; Gabor Szabo: Funding acquisition; Andreas Simm: Resources, Writing - Review & Editing, Funding acquisition.

## Declaration of generative AI and AI-assisted technologies in the writing process

During the preparation of this work, the authors used Grammarly for Microsoft Office (Ver. 6.8.261, use date June 05, 2025) and ChatGPT (model: GPT-4-turbo, use date February 06, 2025) to improve grammar, language and readability. After using this tool/service, the authors reviewed and edited the content as needed and take full responsibility for the content of the publication.

## Funding

This research was funded by the 10.13039/501100001659German Research Foundation (RTG 2155 ProMoAge).

K.W. was funded by the 10.13039/501100000780European Union (ERDF-European Regional Development Fund), and the State of Saxony-Anhalt, Germany (Autonomy in old Age (AiA), ID: ZS/2018/12/96224).

## Declaration of competing interest

The authors declare the following financial interests/personal relationships which may be considered as potential competing interests: Andreas Simm reports financial support was provided by 10.13039/501100001659German Research Foundation. Merve Kuru-Schors reports financial support was provided by 10.13039/501100001659German Research Foundation. Kristin Waechter reports financial support was provided by European Regional Development Fund. If there are other authors, they declare that they have no known competing financial interests or personal relationships that could have appeared to influence the work reported in this paper.

## Data Availability

Data will be made available on request.

## References

[bib1] Abudahab S., Price E.T., Dozmorov M.G., Deshpande L.S., McClay J.L. (2023). The aryl hydrocarbon receptor, epigenetics and the aging process. J. Nutr. Health Aging.

[bib2] Bahman F., Choudhry K., Al-Rashed F., Al-Mulla F., Sindhu S., Ahmad R. (2024). Aryl hydrocarbon receptor: current perspectives on key signaling partners and immunoregulatory role in inflammatory diseases. Front. Immunol..

[bib3] Bartling B., Rehbein G., Somoza V., Silber R.E., Simm A. (2005). Maillard reaction product‐rich food impair cell proliferation and induce cell death in vitro. Signal Transduct..

[bib4] Bartling B., Fuchs C., Somoza V., Niemann B., Silber R.E., Simm A. (2007). Lung level of HMBG1 is elevated in response to advanced glycation end product-enriched food. Mol. Nutr. Food Res..

[bib5] Behrens A., Schirmer K., Bols N.C., Segner H. (1998). Microassay for rapid measurement of 7-ethoxyresorufin-O-deethylase activity in intact fish hepatocytes. Mar. Environ. Res..

[bib6] Chan L.P., Liu C., Chiang F.Y., Wang L.F., Lee K.W., Chen W.T. (2017). IL-8 promotes inflammatory mediators and stimulates activation of p38 MAPK/ERK-NF-kappaB pathway and reduction of JNK in HNSCC. Oncotarget.

[bib7] Chapkin R.S., Davidson L.A., Park H., Jin U.H., Fan Y.Y., Cheng Y. (2021). Role of the aryl hydrocarbon receptor (AhR) in mediating the effects of coffee in the Colon. Mol. Nutr. Food Res..

[bib8] De Juan A., Segura E. (2021). Modulation of immune responses by nutritional ligands of aryl hydrocarbon receptor. Front. Immunol..

[bib9] Denison M.S., Faber S.C. (2017). And now for something completely different: diversity in ligand-dependent activation of Ah receptor responses. Curr Opin Toxicol.

[bib10] Frejno M., Zenezini Chiozzi R., Wilhelm M., Koch H., Zheng R., Klaeger S. (2017). Pharmacoproteomic characterisation of human Colon and rectal cancer. Mol. Syst. Biol..

[bib11] Geiger T., Wehner A., Schaab C., Cox J., Mann M. (2012). Comparative proteomic analysis of eleven common cell lines reveals ubiquitous but varying expression of most proteins. Mol. Cell. Proteomics.

[bib12] Grosskopf H., Walter K., Karkossa I., von Bergen M., Schubert K. (2021). Non-genomic AhR-Signaling modulates the immune response in endotoxin-activated macrophages after activation by the environmental stressor BaP. Front. Immunol..

[bib13] Heilmann M., Wellner A., Gadermaier G., Ilchmann A., Briza P., Krause M. (2014). Ovalbumin modified with pyrraline, a maillard reaction product, shows enhanced T-cell immunogenicity. J. Biol. Chem..

[bib14] Hellwig M., Geissler S., Peto A., Knütter I., Brandsch M., Henle T. (2009). Transport of free and peptide-bound pyrraline at intestinal and renal epithelial cells. J. Agric. Food Chem..

[bib15] Hellwig M., Diel P., Eisenbrand G., Grune T., Guth S., Henle T. (2024). Dietary glycation compounds - implications for human health. Crit. Rev. Toxicol..

[bib16] Ishikawa T., Takahashi S., Morita K., Okinaga H., Teramoto T. (2014). Induction of AhR-mediated gene transcription by coffee. PLoS One.

[bib17] Jansen F.A.C., Fogliano V., Rubert J., Hoppenbrouwers T. (2023). Dietary advanced glycation end products interacting with the intestinal epithelium: what do we really know?. Mol. Metabol..

[bib18] Jin H., Zhang C., Zwahlen M., von Feilitzen K., Karlsson M., Shi M. (2023). Systematic transcriptional analysis of human cell lines for gene expression landscape and tumor representation. Nat. Commun..

[bib19] Jost T., Henning C., Heymann T., Glomb M.A. (2021). Comprehensive analyses of carbohydrates, 1,2-Dicarbonyl compounds, and advanced glycation end products in industrial bread making. J. Agric. Food Chem..

[bib20] Korca E., Piskovatska V., Borgermann J., Navarrete Santos A., Simm A. (2020). Circulating antibodies against age-modified proteins in patients with coronary atherosclerosis. Sci. Rep..

[bib21] Korkmaz-Icöz S., Schwär M., Loganathan S., Wächter K., Georgevici A.-I., Kraft P. (2022). Bread crust extract protects rats' vascular grafts from in vitro ischemia/reperfusion injury. J. Funct.Foods.

[bib22] Lee W.-J., Liu S.-H., Chiang C.-K., Lin S.-Y., Liang K.-W., Chen C.-H. (2016). Aryl hydrocarbon receptor deficiency attenuates oxidative stress stress-related mesangial cell activation and macrophage infiltration and extracellular matrix accumulation in diabetic nephropathy. Antioxidants Redox Signal..

[bib23] Leuner B., Ruhs S., Brömme H.J., Bierhaus A., Sel S., Silber R.E. (2012). RAGE-Dependent activation of gene expression of superoxide dismutase and vanins by AGE-rich extracts in mice cardiac tissue and murine cardiac fibroblasts. Food Funct..

[bib24] Liu R., Yang Y.H., Cui X.J., Mwabulili F., Xie Y.L. (2023). Effects of baking and frying on the protein oxidation of wheat dough. Foods.

[bib25] Mackowiak B., Wang H. (2016). Mechanisms of xenobiotic receptor activation: direct vs. indirect. Biochim. Biophys. Acta.

[bib26] Medina M.V., Sapochnik D., Garcia Sola M., Coso O. (2020). Regulation of the expression of Heme Oxygenase-1: signal transduction, gene promoter activation, and beyond. Antioxidants Redox Signal..

[bib27] Milkovska-Stamenova S., Hoffmann R. (2019). Diversity of advanced glycation end products in the bovine milk proteome. Amino Acids.

[bib28] Morales F.J., Somoza V., Fogliano V. (2012). Physiological relevance of dietary melanoidins. Amino Acids.

[bib29] Nogueira Silva Lima M.T., Delayre-Orthez C., Howsam M., Jacolot P., Niquet-Leridon C., Okwieka A. (2024). Early- and life-long intake of dietary advanced glycation end-products (dAGEs) leads to transient tissue accumulation, increased gut sensitivity to inflammation, and slight changes in gut microbial diversity, without causing overt disease. Food Res. Int..

[bib30] Ondrova K., Zuvalova I., Vyhlidalova B., Krasulova K., Mikova E., Vrzal R. (2023). Monoterpenoid aryl hydrocarbon receptor allosteric antagonists protect against ultraviolet skin damage in female mice. Nat. Commun..

[bib31] Opitz C.A., Holfelder P., Prentzell M.T., Trump S. (2023). The complex biology of aryl hydrocarbon receptor activation in cancer and beyond. Biochem. Pharmacol..

[bib32] Ott C., Jacobs K., Haucke E., Navarrete Santos A., Grune T., Simm A. (2014). Role of advanced glycation end products in cellular signaling. Redox Biol..

[bib33] Pötzsch S., Dalgalarrondo M., Bakan B., Marion D., Somoza V., Silber R.E. (2013). PP54 - identification of gliadin as an advanced glycation end product-modified compound in bread crust extract and their effect on mouse macrophage activation. Free Radic. Biol. Med..

[bib34] Pötzsch S., Blankenhorn A., Santos A.N., Silber R.E., Somoza V., Simm A. (2013). The effect of an AGE-rich dietary extract on the activation of NF-κB depends on the cell model used. Food Funct..

[bib35] Poulsen M.W., Hedegaard R.V., Andersen J.M., de Courten B., Bügel S., Nielsen J. (2013). Advanced glycation endproducts in food and their effects on health. Food Chem. Toxicol..

[bib36] Rabbani N., Thornalley P.J. (2015). Dicarbonyl stress in cell and tissue dysfunction contributing to ageing and disease. Biochem. Biophys. Res. Commun..

[bib37] Raupbach J., Muller S.K., Schnell V., Friedrich S., Hellwig A., Grune T. (2023). The effect of free and protein-bound maillard reaction products N-epsilon-Carboxymethyllysine, N-epsilon-Fructosyllysine, and pyrraline on Nrf2 and NFkappaB in HCT 116 cells. Mol. Nutr. Food Res..

[bib38] Reed L., Jarvis I.W.H., Phillips D.H., Arlt V.M. (2020). Enhanced DNA adduct formation by benzo[a]pyrene in human liver cells lacking cytochrome P450 oxidoreductase. Mutat. Res. Genet. Toxicol. Environ. Mutagen.

[bib39] Reichard J.F., Motz G.T., Puga A. (2007). Heme oxygenase-1 induction by NRF2 requires inactivation of the transcriptional repressor BACH1. Nucleic Acids Res..

[bib40] Ruhs S., Nass N., Somoza V., Friess U., Schinzel R., Silber R.E. (2007). Maillard reaction products enriched food extract reduce the expression of myofibroblast phenotype markers. Mol. Nutr. Food Res..

[bib41] Scheijen J., Clevers E., Engelen L., Dagnelie P.C., Brouns F., Stehouwer C.D.A. (2016). Analysis of advanced glycation endproducts in selected food items by ultra-performance liquid chromatography tandem mass spectrometry: presentation of a dietary AGE database. Food Chem..

[bib42] Sebekova K., Faist V., Hofmann T., Schinzel R., Heidland A. (2003). Effects of a diet rich in advanced glycation end products in the rat remnant kidney model. Am. J. Kidney Dis..

[bib43] Seok S.H., Ma Z.X., Feltenberger J.B., Chen H., Chen H., Scarlett C. (2018). Trace derivatives of kynurenine potently activate the aryl hydrocarbon receptor (AHR). J. Biol. Chem..

[bib44] Sergi D., Boulestin H., Campbell F.M., Williams L.M. (2021). The role of dietary advanced glycation end products in metabolic dysfunction. Mol. Nutr. Food Res..

[bib45] Shi J., Wang X., Lyu L., Jiang H., Zhu H.J. (2018). Comparison of protein expression between human livers and the hepatic cell lines HepG2, Hep3B, and Huh7 using SWATH and MRM-HR proteomics: focusing on drug-metabolizing enzymes. Drug Metabol. Pharmacokinet..

[bib46] Shiromizu T., Adachi J., Watanabe S., Murakami T., Kuga T., Muraoka S. (2013). Identification of missing proteins in the neXtProt database and unregistered phosphopeptides in the PhosphoSitePlus database as part of the Chromosome-centric human proteome project. J. Proteome Res..

[bib47] Sladekova L., Mani S., Dvorak Z. (2023). Ligands and agonists of the aryl hydrocarbon receptor AhR: facts and myths. Biochem. Pharmacol..

[bib48] Snelson M., Coughlan M.T. (2019). Dietary advanced glycation end products: digestion, metabolism and modulation of gut microbial ecology. Nutrients.

[bib49] Toydemir G., Loonen L.M.P., Venkatasubramanian P.B., Mes J.J., Wells J.M., De Wit N. (2021). Coffee induces AHR- and Nrf2-mediated transcription in intestinal epithelial cells. Food Chem..

[bib50] Twarda-Clapa A., Olczak A., Bialkowska A.M., Koziolkiewicz M. (2022). Advanced glycation end-products (AGEs): formation, chemistry, classification, receptors, and diseases related to AGEs. Cells.

[bib51] Uceda A.B., Marino L., Casasnovas R., Adrover M. (2024). An overview on glycation: molecular mechanisms, impact on proteins, pathogenesis, and inhibition. Biophys. Rev..

[bib52] van Dongen K.C.W., Kappetein L., Estruch I.M., Belzer C., Beekmann K., Rietjens I.M.C.M. (2022). Differences in kinetics and dynamics of endogenous versus exogenous advanced glycation end products (AGEs) and their precursors. Food Chem. Toxicol..

[bib55] Wächter K., Longin C.F.H., Winterhalter P.R., Bertsche U., Szabo G., Simm A. (2023). The antioxidant potential of various wheat crusts correlates with AGE content independently of acrylamide. Foods.

[bib54] Wächter K., Gohde B., Szabó G., Simm A. (2022). Rye bread crust as an inducer of antioxidant genes and suppressor of NF-κB pathway in vivo. Nutrients.

[bib53] Wächter K., Navarrete Santos A., Grosskopf A., Baldensperger T., Glomb M.A., Szabo G. (2021). AGE-rich bread crust extract boosts oxidative stress interception via stimulation of the NRF2 pathway. Nutrients.

[bib56] Ye W., Chen R., Chen X., Huang B., Lin R., Xie X. (2019). AhR regulates the expression of human cytochrome P450 1A1 (CYP1A1) by recruiting Sp1. FEBS J..

[bib57] Yilmaz C., Gokmen V. (2018). Determination of tryptophan derivatives in kynurenine pathway in fermented foods using liquid chromatography tandem mass spectrometry. Food Chem..

[bib58] Zhang Y.C., Albrecht D., Bomser J., Schwartz S.J., Vodovotz Y. (2003). Isoflavone profile and biological activity of soy bread. J. Agric. Food Chem..

[bib59] Zhang Q.Z., Li H.T., Zheng R.X., Cao L.L., Zhang S.F., Zhang S.F. (2024). Comprehensive analysis of advanced glycation end-products in commonly consumed foods: presenting a database for dietary AGEs and associated exposure assessment. Food Sci. Hum. Wellness.

