# Supplementary material for: Bread crust extract is a novel activator of aryl hydrocarbon receptor and modulator of NRF2 and NFκB in HepG2 and HCT 116 cells
Source: Curr Res Food Sci. 2025 Jul 12;11:101144. doi: 10.1016/j.crfs.2025.101144 (PMC12284547; doi:10.1016/j.crfs.2025.101144)
Supplement: Multimedia component 1 [file mmc1.docx]

Bread Crust Extract is a Novel Activator of Aryl Hydrocarbon Receptor and Modulator of NRF2 and NFκB in HepG2 and HCT116 cells.

**Supplemental Materials and Methods**

*Chemicals and antibodies*

6-Formylindolo[3,2-b]carbazole (FICZ) was purchased from Hycultec (HY-12451, Beutelsbach, Germany). Tert-Butylhydroquinone (tBHQ, 112941), L-kynurenine (K8625), benzo-a-pyrene (BaP, B1760), chloroform puriss.p.a. (32211-1L-M), isopropanol (33539-M), Triton X-100 (X-100), 1x Protease inhibitor cocktail (P8340), sodium orthovanadate (S6508) and sodium deoxycholate (D6750) were purchased from Merck / Sigma-Aldrich (St. Louis, MO, USA). Bright-Glo™ Luciferase Assay System (E2620) and Cell Titer-Blue reagent (G8080) were from Promega (Madison, WI, USA). Quanti-Luc Gold (rep-qlcs-5, rep-qlcg5), Zeocin (ant-zn-1), Normocin (ant-nr-2), and Hygromycin B Gold (ant-hg-1) were from InvivoGen (San Diego, CA, USA).

McCoy's 5A medium (16600082), DMEM with 4.5g/L glucose (41966-029), Phenol red-free DMEM (31053-028), PBS, 10,000 U/ml Penicillin-Streptomycin solution (11548876), hygromycin B solution, 200 mM L-glutamine (15430614), GlutaMAX supplement (13462629), 0.5% Trypsin/EDTA solution (10779413), and TRIzol reagent (Invitrogen™, 15596018) were from Thermo Fisher Scientific (Waltham, MA, USA).

Dicoumarol (Cay20764-1) and 7-Ethoxyresorufin (Cay16122-1) were produced by Cayman Chemical and distributed by Biomol (Hamburg, Germany).

All Maillard reaction products were purchased from Iris Biotech (Marktredwitz, Germany)

Auranofin was obtained from Enzo Life Sciences (BML-EI206-0100, Lörrach, Germany) and recombinant human tumor necrosis factor alpha (TNF-α) was from PeproTech (300-01A, Hamburg, Germany).

Tris(hydroxymethyl)-aminomethane hydrochloride (Tris-HCl, 812 846) was purchased from Boehringer Mannheim. Sodium chloride (A3597) and bovine serum albumin (BSA) fraction V (A1391) were from AppliChem. Sodium dodecyl sulfate (SDS, 20% stock solution, 1057) was from Carl Roth.

Anti-Aryl hydrocarbon-receptor (AHR) antibody (sc-133088, dilution 1:150), anti-Kynurenine antibody (sc-69890, dilution 1:200), and anti-benzo[a]pyrene antibody (sc-51508, dilution 1:200) were from Santacruz Biotechnology. Goat-anti-mouse conjugated secondary antibody IRDye 800CW, Isotype IgG, diluted 1:10,000, was purchased from LI-COR Biotechnology (926-32212).

*Slot Blot*

For slot blot analysis, BCE, R-HSA and HSA were diluted in PBS and applied onto a 0.2 µm Nitrocellulose membrane via the Minifold Blotting device (Schleicher & Schuell Whatman, London, UK) attached to a water-jet vacuum pump. After rinsing two times with PBS, the membrane was removed and blocked in 5% BSA in TBS. Subsequently, antibody detection was carried out as described for western blotting.

*Cell viability and metabolic activity*

For determination of toxic concentrations and control of abnormalities during the assay, Cell Titer Blue (CTB) reagent (Promega, G8081) was used according to the manufacturer’s protocol. In short, cells were washed with PBS and then 100 µl of fresh assay medium and 100 µl of CTB were added, shaken for 10s, and then incubated for 2 h at 37°C, 5% CO2. For HepG2-AHR, the same plate as for the experiment was utilized for the CTB. For the other reporter lines, a parallel plate was prepared. Fluorescent measurements were carried out at the CLARIOstar plus device (Table S1).

Table S1: Measurement settings for reporter luminescence and CTB. Bw: bandwidth

|  | AHR reporter | NRF2/ARE and  NFκB reporter | CTB |
| --- | --- | --- | --- |
| mode | luminescence | luminescence | fluorescence |
| type | endpoint | endpoint | endpoint |
| optics | top | top | top |
| reset | Renilla | Firefly | Resorufin |
| Ex., bw | - | - | 545 nm, 20 nm |
| Em, bw | 480nm, 80 nm | - | 600 nm, 40 nm |
| gain | fixed: 3600 | fixed: 3600 | - |
| focal height | 7 mm | 11.5 mm | - |
| settling time | 1 s | - | 0.5 s |
| interval time | 0.1 s | 1s | - |
| dynamic range | - | enhanced | - |
| aperture | installed | installed | - |
| flashes | - | - | 20 |

*qRT-PCR*

Table S2: qPCR Primer information. NM_numbers denote the RefSeq entries in which the primers bind. If more than one NM is given, several transcript variants of the same target are detected. For: forward primer, rev: reverse primer, bp: base pairs. # for the SOD2 primer, another predicted target is reported by Primer-BLAST, but with >4 mismatches and a size > 2500 bp. * The RAGE primer potentially detects two targets in the RAGE mRNA, but the 278 bp product is not detected in the used conditions.

| target name | NM accessions(s) | Primer sequences (5'->3') | amplicon length (bp) | source |
| --- | --- | --- | --- | --- |
| **RPLP0** | NM_053275.4  NM_001002.4 | for: TCGACAATGGCAGCATCTAC  rev: GCCTTGACCTTTTCAGCAAG | 223 |  |
| **CYP1A1** | NM_000499.5  NM_001319217.2 | for: CAAGGGGCGTTGTGTCTTTG  rev: GTCGATAGCACCATCAGGGG | 115 |  |
| **SOD2**  **(Mn-SOD)** | NM_001322819.2  NM_001322815.2  NM_001322817.2  NM_001024465.3  NM_001322820.2  NM_000636.4 # | for: CACCGAGGAGAAGTACCAGG  rev: TAGGGCTGAGGTTTGTCCAG | 131 | 10 |
| **CXCL8 (IL-8)** | NM_001354840.3  NM_000584.4 | for: ACACTGCGCCAACACAGAAA  rev: CAACCCTCTGCACCCAGTTT | 89 |  |
| **HMOX1** | NM_002133.3 | for: CCAGCAACAAAGTGCAAGAT  rev: CATTCACATGGCATAAAGCC | 152 |  |

Table S3: Quality controls for quantitative real time PCR primers.

|  | **NTC**  **HCT 116 (Cq)** | **NTC**  **HepG2 (Cq)** | **efficiency** | **LOQ** | **LOD** |
| --- | --- | --- | --- | --- | --- |
| **RPLP0** | 35.39 | 34.71 | 1.99 | 0.4 ng | 0.04 ng |
| **CYP1A1** | 39.20 | 39.92 | 1.98 | 0.04 ng | 0.004 ng |
| **IL-8** | 34.88 | 36.01 | 1.96 | 0.4 ng | 0.04 ng |
| **HMOX1** | - | - | 2.03 | 0.04 ng | 0.004 ng |
| **Mn-SOD** | 39.34 | 36.61 | 2.04 | 0.4 ng | 0.04 ng |

Table S4: qRT-PCR run settings

| **step** | **time** | **temperature** |
| --- | --- | --- |
| activation and denaturation | 30 s | 95 °C |
| 40 x amplification  denaturation  annealing & extension | 15 s  30 s | 95 °C  62 °C |
| hold |  | 10 °C |
| Melt curve | 0.5 s increments | 0.5 °C, 65 – 95 °C |

Fig. S1. Activation of HepG2 NFκB reporter cells by increasing concentrations of TNFα to determine maximal activation of the reporter construct after 24h (N=2, n=3). Stimulation with 20 ng/ml TNFα led to only sub-maximal reporter activation.

Fig. S2. Serial dilutions of cDNA input for primer quality control and efficiency determination.

**Supplemental Results**

Table S5. Parameters from agonist vs. response (three parameters) non-linear fit of the EC_50_ for BCE in HepG2-AHR reporter cells. CI: confidence interval

|  | **24 h** | **48 h** |
| --- | --- | --- |
| **Best-fit values** | | |
| Bottom | 0.9919 | 0.7500 |
| Top | = 8.290 | = 20.63 |
| EC50 | 1.115 | 3.360 |
| logEC50 | 0.04738 | 0.5263 |
| Span | 7.298 | 19.88 |
| **95% CI (profile likelihood)** | | |
| Bottom | 0.2827 to 1.696 | -0.3789 to 1.873 |
| EC50 | 0.5822 to 1.998 | 2.450 to 4.638 |
| logEC50 | -0.2349 to 0.3006 | 0.3892 to 0.6663 |
| **Goodness of Fit** |  |  |
| Degrees of Freedom | 28 | 28 |
| R squared | 0.8072 | 0.8746 |
| Sum of Squares | 55.17 | 153.2 |
| Sy.x | 1.404 | 2.339 |
| **Constraints** | | |
| Top | = 8.290 | = 20.63 |


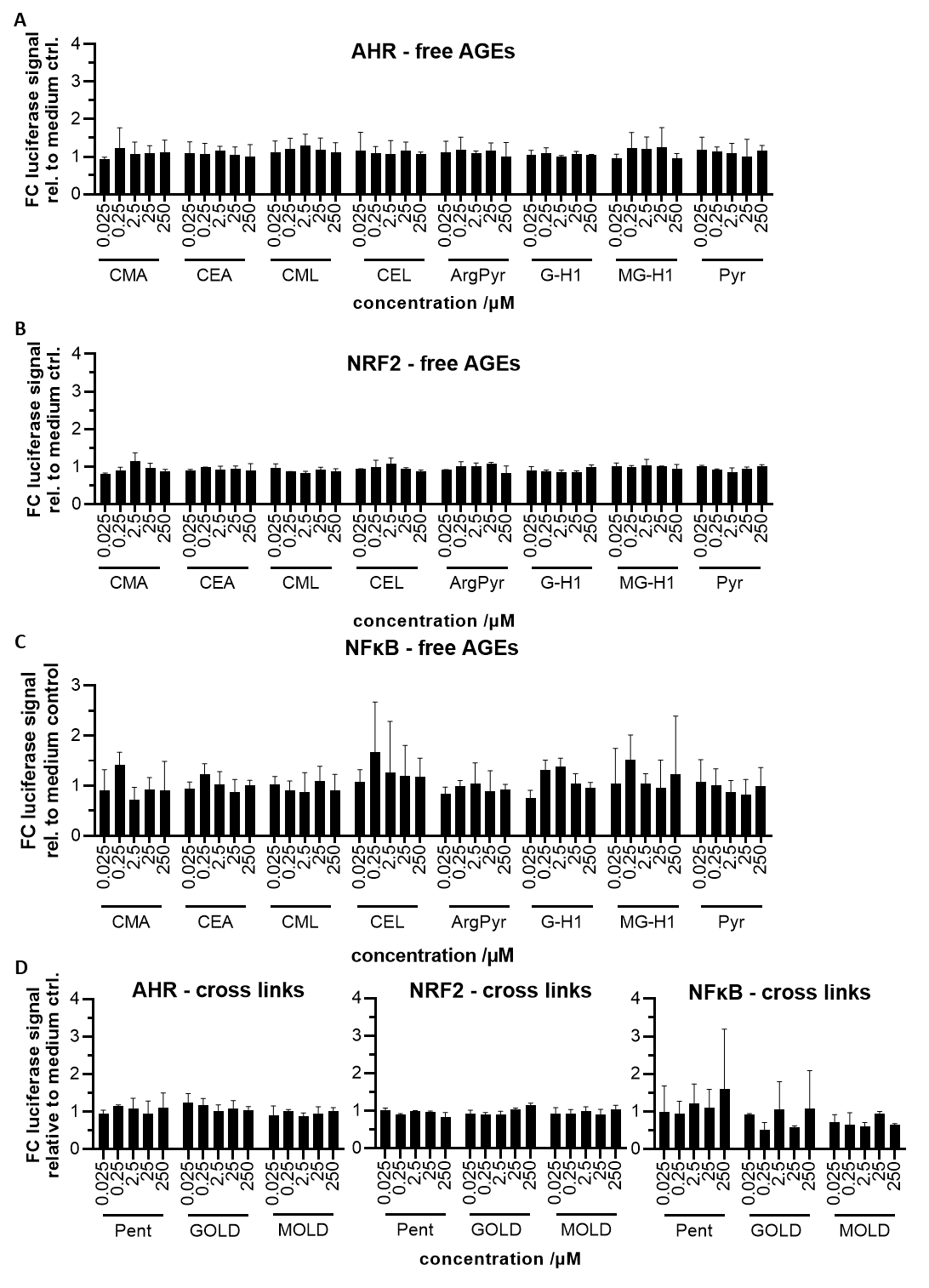


Fig. S3. HepG2 reporter cell response to a concentration range of free AGEs and cross-links after 24h of incubation in mean fold-change (FC) luciferase signal induction above the medium signal ± SD. A value of 1 indicates no change. N=3, n= 3. A & D: HepG2 AHR reporter, B & E: HepG2 NRF2-reporter, C & F: HepG2 NFκB-reporter cells. N-ω-carboxymethyl-L-arginine (CMA), N-ω-carboxyethyl-L-arginine (CEA), N-ε-carboxymethyl-L-Lysine (CML), N-ε-carboxyethyl-L-Lysine (CEL), Argpyrimidine (ArgPyr), Glyoxal-hydroimidazolone isomer (G-H1), Methylglyoxal-hydroimidazolone isomer (MG-H1), and pyrraline (Pyr) and three crosslinks, pentosidine (Pent), Glyoxyl-derived lysine dimer (GOLD), and Methylglyoxyl-derived lysine dimer (MOLD).


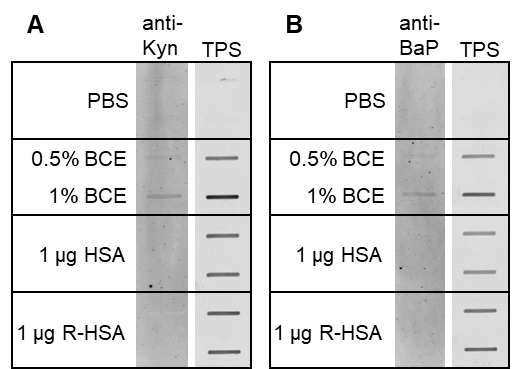


Fig. S4. Antibody-based slot blot detection of kynurenine (Kyn) and benzo[a]pyrene (BaP) in BCE, R-HSA and HSA and total protein stain (TPS) as loading control. A: L-Kyn is detectable in 0.5 and 1 % BCE, but not in 1 µg R-HSA or the controls PBS and HSA. B: BaP is detectable in 0.5 and 1 % BCE, but not in 1 µg R-HSA or the controls PBS and HSA.

Fig. S5. Cell titer blue assay fluorescence normalized to solvent controls show decreased metabolic activity at BaP concentrations > 1 µM in HepG2 AHR, NRF2 and NFκB reporter cells indicating potential cytotoxicity. Zero equals no change while values <0 indicate a decrease.


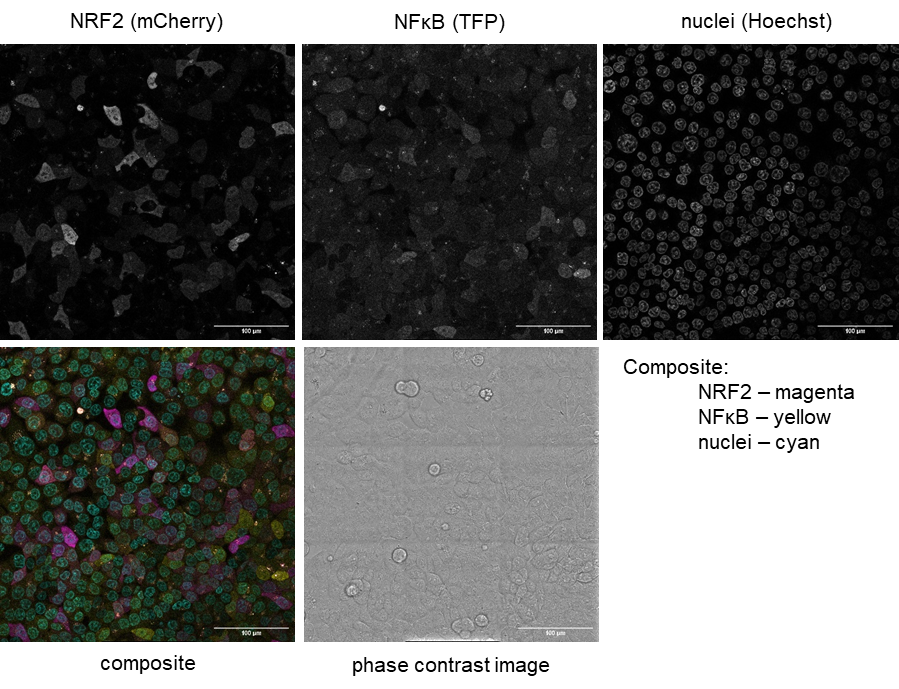


Fig S6. Representative microscopy result for HCT 116 pTRAF reporter. Of each sample, five montages of 9 images were evaluated. One montage of induction by 10% BCE is shown. Images were processed and composite pseudo-colors were chosen for better visibility. Scale bar size is 100 µm.

Table S6. Quantitative data on AGEs in bread varieties (A) in mg AGEs per 100g whole bread from different sources and (B) theoretical calculated amounts in BCE in µM with complete extraction and 94g/L BC, which represents the amount used in extraction protocol.

| **A)**  **mg/100g** | **Zhang et al.**  **(2024)** | | **Scheijen et al.**  **(2016)** | | | | | **Jost et al.**  **(2021)** | | |
| --- | --- | --- | --- | --- | --- | --- | --- | --- | --- | --- |
|  | **whole-meal** | **rye** | **rye a** | **rye b** | **rye**  **light** | **wheat**  **(German)** | **wheat** | | **rye** |  |
| **CMA** |  |  |  |  |  |  | 0.16 | | 0.14 |  |
| **CEA** |  |  |  |  |  |  | 1.5 | | 1.1 |  |
| **CML** | 0.525 | 0.985 | 0.9 | 0.58 | 0.59 | 0.31 | 0.45 | | 0.51 |  |
| **CEL** | 0.53 | 0.137 | 0.09 | 0.18 | 0.18 | 0.13 | 0.23 | | 0.21 |  |
| **G-H1** | 2.376 | 2.066 |  |  |  |  |  | |  |  |
| **MG-H1** | 3.89 | 3.278 | 2.57 | 2.74 | 5.22 | 2.09 | 1.8 | | 1.3 |  |
| **Pyr** |  |  |  |  |  |  | 3.6 | | 2.8 |  |
| **Pent** | 0.412 | 0.226 |  |  |  |  |  | |  |  |
| **GOLD** | 1.517 | 1.428 |  |  |  |  |  | |  |  |
| **MOLD** | 0.391 | 0.344 |  |  |  |  |  | |  |  |
| **B) conc. (µM) in BCE calculated from above (extraction = 100%)** | | | | | | | | | |  |
| **CMA** |  |  |  |  |  |  | 0.65 | | 0.57 |  |
| **CEA** |  |  |  |  |  |  | 0.61 | | 0.45 |  |
| **CML** | 2.42 | 4.53 | 4.14 | 2.67 | 2.72 | 1.43 | 2.07 | | 2.35 |  |
| **CEL** | 2.28 | 0.59 | 0.39 | 0.78 | 0.78 | 0.56 | 0.11 | | 0.10 |  |
| **G-H1** | 10.43 | 9.07 |  |  |  |  |  | |  |  |
| **MG-H1** | 16.02 | 13.50 | 10.58 | 11.28 | 21.50 | 8.61 | 7.41 | | 5.35 |  |
| **Pyr** |  |  |  |  |  |  | 13.31 | | 10.35 |  |
| **Pent** | 1.02 | 0.56 |  |  |  |  |  | |  |  |
| **GOLD** | 4.36 | 4.10 |  |  |  |  |  | |  |  |
| **MOLD** | 1.08 | 0.95 |  |  |  |  |  | |  |  |

Table S7. Result parameters from the one-phase decay analysis with robust regression of AHR presented in Fig. 5 E-H. RSDR: Robust Standard Deviation of the Residuals

|  | **5% BCE** | **150 µM Kyn** | **500 nM BaP** | | **10 µM FICZ** | | |
| --- | --- | --- | --- | --- | --- | --- | --- |
| **Best-fit values** | | | |  | |  |  |
| Y0 | = 1.000 | = 1.000 | = 1.000 | | = 1.000 | | |
| Plateau | 0.3986 | 0.4906 | 0.3659 | | 0.3640 | | |
| K | 0.04199 | 1.214 | 0.3571 | | 1.001 | | |
| Half Life (h) | 16.51 | 0.5711 | 1.941 | | 0.6928 | | |
| Tau | 23.82 | 0.8240 | 2.800 | | 0.9995 | | |
| Span | 0.6014 | 0.5094 | 0.6341 | | 0.6360 | | |
| **Goodness of Fit** | | | |  | |  |  |
| Robust Sum of Squares | 6.240 | 8.181 | 9.688 | | 6.562 | | |
| RSDR | 0.1703 | 0.04331 | 0.05717 | | 0.08971 | | |
| **Constraints** | | | |  | |  |  |
| Y0 | Y0 = 1 | Y0 = 1 | Y0 = 1 | | Y0 = 1 | | |
| K | K > 0 | K > 0 | K > 0 | | K > 0 | | |
